# Exploring the roles of HPV16 variants in head and neck squamous cell carcinoma: current challenges and opportunities

**DOI:** 10.1186/s12985-021-01688-9

**Published:** 2021-11-08

**Authors:** Daniela Cochicho, Rui Gil da Costa, Ana Felix

**Affiliations:** 1 NOVA Medical School, NOVA University of Lisbon, CEDOC, Campo Mártires da Pátria 130, 1169-056 Lisbon, Portugal; 2 Virology Laboratory IPOLFG, Rua Professor Lima Bastos, 1099-023 Lisbon, Portugal; 3 LEPABE, Laboratory for Process Engineering, Environment, Biotechnology and Energy, University of Porto, Rua Dr. Roberto Frias, 4200-465 Porto, Portugal; 4 Post-graduate Programme in Adult Health (PPGSAD), University Hospital (HUUFMA) and Morphology Department, Federal University of Maranhão, Av. dos Portugueses 1966 - Vila Bacanga, São Luís, MA 65080-805 Brazil; 5 Molecular Oncology and Viral Pathology Group, Research Center of IPO Porto (CI-IPOP) / RISE@CI-IPOP (Health Research Network), Portuguese Oncology Institute of Porto (IPO Porto) / Porto Comprehensive Cancer Center (Porto.CCC), Rua Dr. António Bernardino de Almeida, 4200-072 Porto, Portugal; 6 Pathology Department IPOLFG, Rua Professor Lima Bastos, 1099-023 Lisbon, Portugal

**Keywords:** HNSCC, HPV16, HPV16 variants, Next generation sequencing

## Abstract

The incidence of squamous cell carcinomas of the head and neck (HNSCC) is consistently increasing, in association with human papillomavirus (HPV) infection, especially HPV16. HPV variants show heterogeneity in the pathogenicity of cervical cancer, but little has been established about their relevance on HNSCC. This review addresses the distribution of HPV16 variants in HNSCC and their potential contribution to clinical practice. A search was performed in PubMed using the keywords HNSCC HPV16 variants. Sixty articles were identified between 2000 and 2020 and 9 articles were selected for a systematic analysis. Clinical cohorts comprised 4 to 253 patients aged between 17 and 91 years with confirmed HPV16-positive HNSCC. Samples were collected from fresh biopsies of the tumour, oral rinse or formol fixed/paraffin embedded tissue, from the oral cavity, oropharynx, hypopharynx, larynx and Waldeyer's tonsillar ring. HPV16 variants were identified using Sanger sequencing techniques. Seven studies addressed the HPV16 *E*6 gene, one studied *E*6 and *E*7, another studied *L*1 and one focused on the long control region. European variants represent 25–95%, Asian-American 5–57% and African 2–4% of the total isolates, suggesting a marked predominance of European strains. No correlations could be drawn with patient prognosis, partly because many studies relied on small patient cohorts. Additional studies are needed, particularly those employing next generation sequencing techniques (NGS), which will allow faster and accurate analysis of large numbers of samples.

## Background

Head and neck cancer is the sixth leading cancer by incidence worldwide [1] and comprises many different pathological entities. The diagnosis is often characterized by multifocal development and presentation at an advanced stage. [2] The age at diagnosis usually ranges between 50 and 70 years of age and men are significantly more likely to develop the disease than women (up to a 4:1 proportion, depending on geographical localization) [3]. Currently, two major carcinogenic pathways leading to head and neck squamous cell carcinomas are recognized: the first is associated with risk factors like smoking and abusive alcohol consumption while the second is associated with human papillomavirus (HPV) infection [4, 5]. Over the past decade, several epidemiologic studies reported a 36.5% increase in the incidence of head and neck squamous cell carcinomas (HNSCC), particularly in high-income countries and among men < 60 years [4, 6–8]. Available data indicates that these changes specifically involve oropharyngeal cancers [9, 10]. Although tobacco consumption has decreased, the incidence of HPV-positive oropharyngeal cancers has increased [11], indicating that HPV infection is the underlying cause for the overall increase in HNSCC incidence [12–14]. Patients showing oral HPV infection are 53 times more likely to develop HNSCC [15]. Presently, HPV is present in approximately 35% of HNSCC and most HPV-positive cases emerge in lingual and palatine tonsils [16]. The presence of HPV DNA in tumour cells defines a specific pathologic entity within HNSCC with specific epidemiology, molecular characteristics, and biological behaviour [17]. HPV interferes with key signaling pathways to promote carcinogenesis via its viral oncoproteins E6 and E7, which lead to the inactivation of tumour protein 53 (p53) and the retinoblastoma protein (pRB), respectively [18]. HPV is a group of small, double-stranded DNA viruses that infect the epidermis and keratinizing mucosae. More than 100 HPV types have been identified [19, 20]. Presently, 40 different HPV types are known to infect mucosal epithelia and are categorized into low-risk and high-risk HPV types according to their epidemiologic association with cervical cancer [21]. In HNSCC, the majority of HPV types belong to this group including HPV16, HPV18, HPV39 and HPV45 [22]. HPV16 is by far, the most detected type, accounting for 90% of all HPV-positive HNSCC cases [23], a significantly greater proportion than in cervical cancer where it accounts for little over 50% of cases [24]. Sequencing of the HPV genome also revealed intra-type variants with genetic differences ranging between 0.5 and 1% [25, 26]. HPV variants arise mainly from nucleotide substitutions in some restricted positions in the genome coding region or in the noncoding region [27, 28]. The prevalence of variants for each HPV type varies significantly in different geographical areas [27]. Regarding HPV16, whole-genome analysis allowed the characterization of five distinct phylogenetic clusters named according to their original geographical distribution: European (E), Asian (As), Asian-American (AA), African 1 (Af1) and African 2 (Af2) [29–31]. Subsequently, a new branch, North American 1 (NA1) was identified [32, 33]. Currently, emerging epidemiological, etiological, and molecular data suggest that intra-type HPV variants are biologically distinct and may be associated with different risk of cervical cancer progression [28]. I*n vitro* studies using 3D organotypic epithelial cell cultures showed that HPV16 *E6* variants differ in their ability to abolish keratinocyte differentiation and to induce p53 degradation [34–36]. Those experimental results are corroborated by clinical studies on cervical cancer. The T350G (L83V) HPV16 variant is the most frequently found among invasive cervical cancers [21, 37] and has been linked to a higher oncogenic potential than the prototype [38], possibly by facilitating persistent viral infection, a critical factor for cancer development [39–41]. Despite the growing number of studies concerning HPV16 variants on cervical cancer, there are currently very few reports describing their distribution in HNSCC and nothing is established about their impact on the development, treatment response and impact on disease outcome. Nonetheless, HPV16 variants may also play an important role in head and neck carcinogenesis and studying their impact is needed due to the rising incidence of HPV-positive HNSCC [18, 23, 25, 42]. The purpose of this work is to systematically review the distribution of HPV16 variants in HNSCC and to assess the available knowledge concerning their potential contribution for HNSCC pathologic heterogeneity in published studies.

## Main text

### Review compilation data

**Fig. 1:**
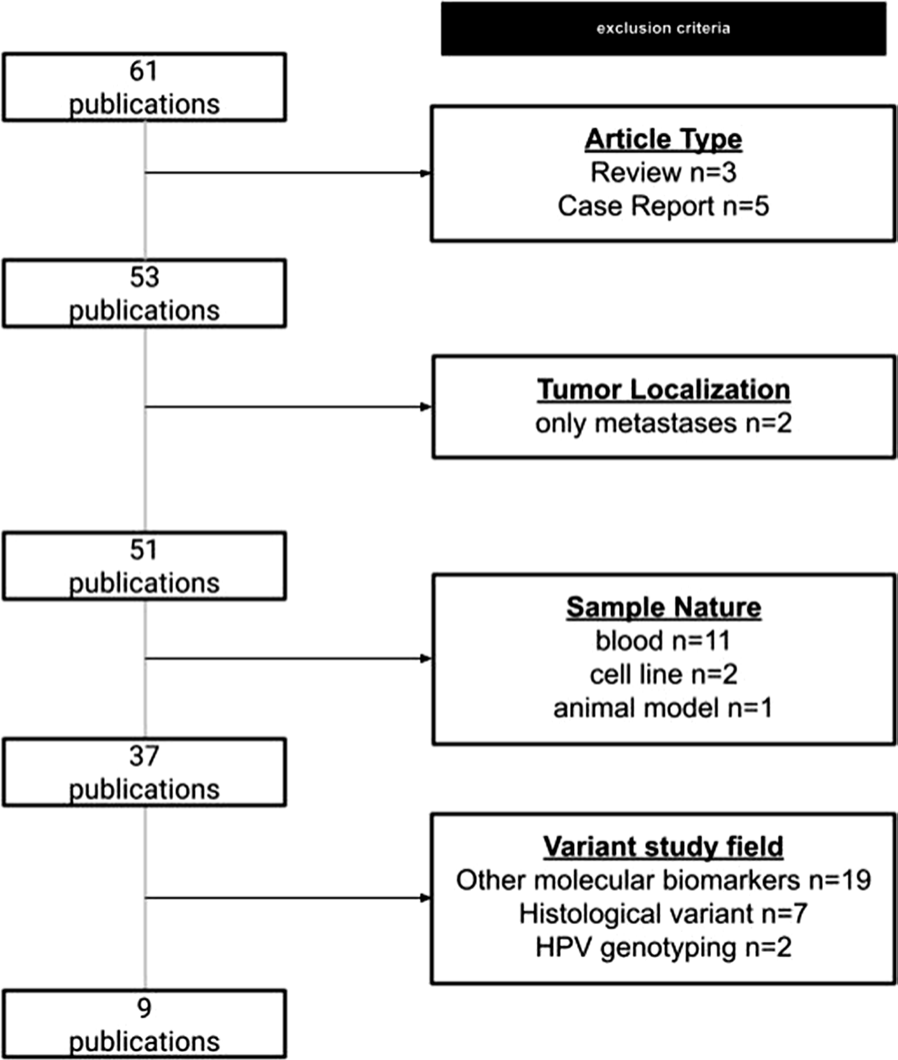
Analytical flow for systematically reviewing published articles dealing with HPV16 variants in HNSCC

**Fig. 2:**
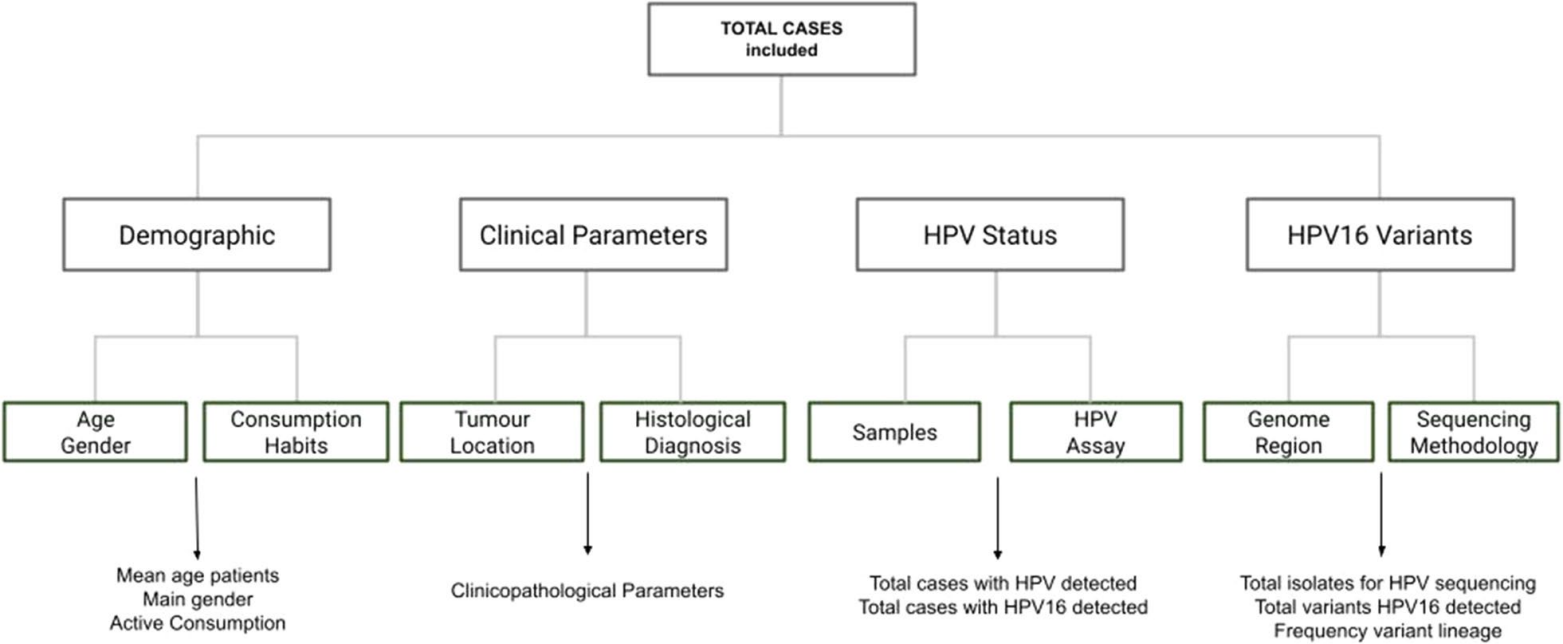
Analytical variables flow for included articles dealing with HPV16 variants in HNSCC search criteria

A systematic review was performed on the PubMed electronic database (https://pubmed.ncbi.nlm.nih.gov) using the following keywords search criteria: HNSCC HPV16 variants. Observational studies reporting the distribution of HPV16 variants in HNSCC and published between 2000 and 2020 were included. Review articles, case reports and articles dealing with other types of cancer were excluded (Fig. 1). The selection search criteria were based on tumour localization, nature of sample and background of the variant study field. The studies were independently assessed to identify the prevalence of HPV16 variants in HNSCC and evaluate in respective cohorts for correlation with clinicopathological parameters. HNSCC patients were categorized into different sites (oral cavity and oropharynx) and further into sub cohorts based on the demographic and clinical information provided. The published data were summarized using frequencies and percentages for HPV16 variants, stratified by tumour location, types of tumour samples, patient cohort size, sequencing methodology, and clinical variables such as age and gender (Fig. 2.)

### General findings

From 61 records found using the search criteria HNSCC HPV16 DNA cell carcinoma variants frequency patients’ cohorts, we selected 9 studies while 52 studies were excluded from further analysis, including review articles and papers which fit the exclusion criteria (Fig. 1). Selected articles report data from a total of 945 patients in the period between 2000 and 2020, distributed by 6 countries. (Table 1) Three studies were performed in Europe, three in the USA, two in Asia and Middle East (Japan and Iran) and one in South America (Brazil). HPV16 variants were evaluated in nine different clinical cohorts. All studies were different and did not contain the evaluation of the same cases. Overall, the study population consisted of patients whose age at diagnosis ranged between 17 and 91 years. The gender ratio was 1:3 or 1:4 (female to male), and all cases were histologically confirmed as HNSCC. The specimens used to evaluate HPV16 were primary tumours located in the oral cavity, oropharynx, hypopharynx, larynx and Waldeyer’s tonsillar ring. Regional lymph node metastases were also included in one study. The samples used to analyze were diverse and consisted of fresh tissue, oral rinse, or formalin fixed/paraffin embedded tissue.

**Table 1:** Identification of HPV16 variants in HNSCC in the literature (PubMed 2000–2020)

Year publication	Reference	Country	Mean age patients (yrs)	Gender (% male)	Consumption (% active)	Samples	Total cases (n)	Total cases HPV detected %(n)	Total cases HPV16 detected %(n)	Total isolates for HPV sequencing	Genome region location	Amplicon size (pb)	Sequencing
2000	[48]	USA	63	no data available	87%	Fresh tissue	253	24% (62)	90% (56)	52	E6	455	Sanger
2004	[49]	Germany	no data available	no data available	no data available	FF/PE	24	100% (24)	100% (24)	21	E6 and E7	793	Sanger
2007	[50]	Italy	63	74%	67%	Fresh tissue FF/PE	115	18% (21)	67% (14/21)	13	L1	150	Sanger
2008	[51]	USA	57	77%	76%	Oral rinse FF/PE	135	32% (44)	100% (44/44)	19	E6	609	Sanger
2009	[52]	Japan	64	86%	79%	Fresh tissue	77	10% (8)	100% (8/8)	8	E6	323	Sanger
2013	[53]	Brazil	59	100%	50%	FF/PE	4	100% (4)	100% (4/4)	4	E6	no data available	Sanger
2015	[54]	Italy	65	88%	63%	FF/PE	24	100% (10)	100% (10/10)	10	E6	no data available	Sanger
2016	[55]	USA	60	86%	No data available	FF/PE	205	18% (36)	70% (25)	21	LCR	193	Sanger
2020	[56]	Iran	56	58%	No data available	FF/PE	108	23% (25)	16% (17)	13	E6	no data available	Sanger

*E6* Early gene 6; *E7* early gene 7; *LCR* long control region; *L1* late gene 1; *FF/PE* Formalin fixed Paraffin embedded

The use of the p16INK4a marker is to clinically detect an oncogenically active HPV infection and is considered as a surrogate marker for HPV infection [43, 44]. From the 9 studies evaluated three described the expression pattern of p16INK4a among negative HNSCC HPV(−) and HNSCC HPV16 cases. For all studies there was a significant difference in p16INK4a expression between HNSCC HPV(−) and HNSCC HPV16(+). Pakdel et al. 2020 observed the expression of p16INK4a among HPV variants and showed that a strong p16INK4a expression was present in poorly differentiated tumour tissues infected with HPV16 sublineage A2 [45].

### HPV detection and genotyping

HPV was detected using PCR-based techniques employing consensus degenerate primers: MY09/11 (4 studies) MY09/11 and/or GP05/06 (2 studies), PGMY (one study) and SPF1/2 (one study); all of which are complementary to the conserved *L1* region. One study detected HPV using in situ hybridization. HPV genotyping was performed using different methods. Two studies using a commercial kit (INNO LIPA) which identifies 28 different HPV types (6, 11, 16, 18, 26, 31, 33, 35, 39, 40, 43, 44, 45, 51, 52, 53, 54, 56, 58, 59, 66, 68, 69, 70, 71, 73, 74, 82) by reverse blot hybridization. Two other studies used TaqMan PCR methods targeting the *E6* or *E6*/LCR. One study used restriction fragment length polymorphism (RFLP) analysis. Two studies performed direct Sanger sequencing of the PCR products. The HPV frequency in the different cohorts ranged from 10 to 100% of the cases, and the HPV16 distribution ranged between 67 and 100% of all HPV positive cases (Table 1). Except the study done in 2020 in Iran, where just 16% of the HPV detected were HPV16. The most common HPV in their series was HPV16 but also followed by HPV18 and HPV11 and the cases were almost located in Oral cavity and Larynx; only 6 tonsils in all 108 cases were evaluated, explaining the low HPV frequency overall.

### Identification HPV16 variants

Of the 9 studies, seven studies identified HPV16 variants by sequencing the *E*6 gene, one addressed variants in the *L*1 gene and another the long control region. In one study the *E*7 gene was also sequenced along with *E*6 (Table 1). In all studies, the identification of HPV16 variants was performed by Sanger sequencing techniques and by comparison to the reference sequence (prototype). Next generation sequencing (NGS) was not used in any of the studies evaluated. However, the reliance on conventional Sanger sequencing is likely to limit the number of cases that can be analyzed and the reliability of results. [26, 46, 47] Sanger sequencing techniques show limitations concerning the size of amplicons, which may compromise the assay sensitivity. Additionally, to overcome polymerase errors and guarantee the integrity and robustness of the results, it is necessary to perform replicates for each case or vector molecular assays [48]. Such replicates add significantly to the workload and costs and constitute a limitation to analyze large patient cohorts. In fact, only two out of nine studies included over 200 HNSCC patients and none analyzed more than 52 HPV-positive patients for HPV16 variants. Future studies aiming to study larger patient cohorts are likely to benefit from NGS techniques, which provide faster and reliable sequencing results of larger amplicons [25, 26, 46].

### Prevalence of HPV16 variants

The frequency of each HPV16 variant (E, NA, AF, AS and AA) was estimated for each of the 9 studies included in the systematic review (Table 2). Gillison et al. [37] first reported data concerning the distribution of HPV16 E6 variants in HNSCC. The authors evaluated 52 HPV16-positive patients from an overall HNSCC cohort comprising 259 patients. The age at diagnosis ranged between 17 and 91 years-old (median, 63 years-old) and the majority were smokers, with or without alcohol consumption (87%). Tumours were localized in the nasopharynx (n = 2), oral cavity (n = 84), oropharynx (n = 60), hypopharynx (n = 21), larynx (n = 86). HPV-positive patients showed significantly improved disease outcomes compared with HPV-negative patients. All samples used were HNSCC fresh tumour specimens. The HPV16 variants were classified into the same phylogenetic group as the European prototype in 75% of cases, Asian in 17%, North American in 4.0% and African 1 in 4.0% of cases. Six novel variants not previously reported (E-G315T, E-G315G, E-C395G, E-A478T, E-A132T, Af1-C311, Af1-A389) were also identified. The authors remarked that the distribution of variants in HNSCC resembled that observed in cervical cancer. However, no conclusions were drawn concerning the potential contribution of specific HPV16 *E6* variants for increasing cancer risk or modifying tumour biopathology and prognosis. Four years later, Hoffmann et al. [38] identified HPV16 variants in 7 out of 21 tumour specimens of HNSCC. The authors analyzed the *E*6 and *E*7 ORFs. Altogether, the DNA samples carried HPV16 prototype European variant (29%), the European variant T350G (38%) and 33% Euro-German variant (A131G + C712A). Again, no conclusions could be drawn concerning the clinical-pathological relevance of the HPV16 variants identified, partly because of the cohort's small size. Badaracco et al. [49], published molecular analysis data on the HPV16 *L1* ORF, from an Italian cohort (total n = 115, of which only 13 were tested for HPV variants), composed of 86 men and 29 women, with a mean age of 63.21 years old. A high percentage of patients were smokers (67%) and alcohol drinkers 43%. Tumours were localized mostly in the oral cavity (n = 60), followed by the larynx (n = 30), the oropharynx (n = 10), the tonsil area (n = 8), the hypopharynx (n = 5) and the sinus/nose (n = 2). In this study, the presence of HPV was not significantly associated with disease-free survival at 2 years. Sixty nine percent of the cases (13 cases) analyzed showed European-German variants (9 cases), 15% were African type 2 (2 cases), 8% Asian-American (1 case), and the remaining 8% (1 case) had an unclassified variant. The predominance of the European variant was unsurprising considering the Italian origin of the patients. No correlations were drawn between the presence of HPV16 variants and any epidemiological, pathological, or clinical data. Agrwal et al. [50], studied 19 HPV16 isolates from a universe of 135 HNSCC samples. The median age at diagnosis was 57 years-old, 77% of the patients were men, most patients had a history of smoking (n = 62 smokers versus 51 non-smokers) but were non-drinkers (73 non-drinkers versus 41 drinkers). All analyzed cases showed European variants and a single case carried an Asian variant. The most common European variant was E-350 T (n = 6), followed by E-350G (n = 4) and E-T131G (n = 2). Importantly, eight of the 19 isolates contained European variants with sequences unique to a single individual. Boscolo-Rizzo et al. [51] analyzed HPV E6 variants in a short (n = 8) case series. The authors showed the presence of the T350G mutation in 5 cases located in the oral cavity and oropharynx, while three tumours located in the larynx and hypopharynx contained HPV 16 prototype sequences. Joseph et al. [52] compared HPV16 variants present in four patients with bilateral tonsillar HNSCC. Two cases carried European variants while two others carried Asian-American variants. The results show that, in all 4 patients, the same HPV16 variant was present in the bilateral tumours, supporting the hypothesis that a single HPV infection, rather than independent infections with distinct agents, is responsible for those bilateral tumours. Hassani et al. [53] studied the HPV16 variants present in 10 cases of tonsillar HNSCC. The authors found that the E-350G-variant was present in 80% of cases while the European prototype was identified in the other 20%. Again, this short case series did not provide data concerning the pathobiological relevance of HPV16 variants. In the following year, Betiol et al. [54] reported the distribution of HPV16 variants in a Brazilian cohort of 21 HNSCC patients. The authors analyzed the HPV16 LCR and found that 12 (57.1%) patients carried European and 9 carried Asian-American (42.9%) variants. The authors remarked that the slight predominance of European variants accompanied observation from their normal cervical samples, suggesting that the distribution of HPV16 variants reflects the overall frequency in each studied population. The most recent study was published by Pakdel et al. [45]. Thirteen HNSCC tissue specimens tested positive for HPV16 using overlapping PCR assays and were analyzed for the presence of *E6* variants. There was a marked predominance of European variants (84.6%) followed by Asian-American (15.4%) variants.

**Table 2:** Frequency of HPV16 variants

Reference	Total cases HPV16 detected (*n*)	Total isolates HPV sequencing (*n*)	HPV16 variant region location	European (E)		North American	African	Asian	Asia American
				All lineages (*n*)	E-350-G (*n*)	(NA) (*n*)	(AF1|AF2) (*n*)	(AS) (*n*)	(AA) (*n*)
[48]	56	52	E6	39	6	2	2	9	0
[49]	24	21	E6 E7	15	8	0	0	0	0
[50]	14	13	L1	9	0	1	2	0	1
[51]	44	19	E6	18	4	0	0	1	0
[52]	8	8	E6	5	5	0	0	0	0
[53]	4	4	E6	2	1	0	0	0	2
[54]	10	10	E6	8	8	0	0	0	0
[55]	25	21	LCR	9	–	0	0	0	12
[56]	17	13	E6	11	–	0	0	0	2

(–) not evaluated; *AA* Asia-American HPV16 Variant; *AF* African HPV16 Variant; *AS* Asian HPV16 variant; *E* European HPV16 variant; *E6* HPV Early protein 6; *E7* HPV Early protein 7; *LCR* long control region; *L1* late gene; *NA* North American HPV16 Variant

## Discussion

We reviewed the published literature on HPV16 variants in HNSCC, aiming to evaluate the biological meaning of those variants in this particular location. The clinical management of HNSCC improved greatly since the recognition of HPV-positive and HPV-negative lesions. Although tumour recurrence still occurs in 10–20% of HPV-positive HNSCC patients, the majority of these patients clearly benefit from therapeutic de-escalation [55]. The lack of adequate biomarkers to define more tailored approaches is still necessary in SCC HPV associated tumours [56]. The large majority of these tumours are associated with HPV16 and a deeper understanding of the bio pathological implications of distinct HPV16 variants would contribute to the molecular characterization of HPV-positive HNSCC and may help to define patient’s subgroups that would benefit from specific therapeutic approaches. Data from cervical cancer patients showed an association between the presence of non-European HPV16 lineages, a longer viral persistence [57, 58] and an increased risk of developing high-grade cervical intraepithelial neoplasia [59]. Apparently, within individuals, HPV genomes harbour high levels of variability in HPV16 genome sequence upon normal to pre-cancer/cancer [60] revealing several fundamental discoveries and suggesting a paradigm shift from HPV16 as a single viral entity to theorize each HPV16 isolated to be a separate virus with distinct carcinogenic potential [61]. This would imply that within HPV16, the genetic variation partly predicts the risk of pre-cancer and cancer [62]. In particular, specific sublineages (A4, C, D2, and D3) have shown a significantly increased risk compared to the most common A1/A2 sublineages [26] and assured D2, for the strongest risk of cancer within glandular epithelium (adenocarcinomas) [26].

The major data from whole-sequences obtained from individual clinical specimens agrees that the genetic variation occurs more commonly on low-grade or benign HPV16 infections [61] and corrected explained In case–control analyses describing the highest amino acid changing variants HPV16 in the controls throughout the genome with cervix cancer. [62] Therefore, E7 oncogene lacks nonsynonymous (amino acid changing) variants in cervical cancers, suggesting the E7 conservation is admitted for carcinogenicity. [61] The specific conservation of the 98 amino acids of E7, that directly disrupts Rb function, was shown to be crucial for trigger carcinogenesis, owing to be commented as a highly specific target for etiologic and therapeutic research [26].

Presenting data from a cohort study that compares genomic characteristics of HPV associated with cervical versus oropharyngeal tumours using DNA sequence analysis showed no significant differences between distribution in HPV16 variants [24], both presenting major prevalence of the European variant. Instead, the HPV E6 gene amplified from oropharyngeal samples reported over more nonsynonymous mutations, but also for the E7 gene, no differences were found in mutation rates between the two anatomical locations [24, 62]. Notably, the E7 gene is conserved in both locations corroborating the recent findings on cervical cancer studies. The apparent restriction on E7 mutations seems to be present in the oropharynx, as well. Indeed, described all both share common biological features, but the important differences present in HPV-genome may explain their distinct pathophysiological mechanisms and susceptibility to treatment. Nevertheless, the invariability of E7 presents an attractive potential target for therapy at both locations. [24]

In all the studies evaluated in this analysis, the most prevalent variant was the European Variant, which belongs to the European lineage and within this lineage the E-350-G HPV16 variant was the most frequent in majority of the studies sequencing based E6 and/or E7 regions with 29% and 53% respectively, although the cohorts had or not an European origin, (cohorts were from Brazil, United States of America, Japan, Italy and Germany (Table 3).

**Table 3:** Frequency HPV16 Variants by region genome location

Reference	Total number of cases	Total isolates for HPV sequencing	HPV16 variant region location	European		North American (NA)	African (AF1|AF2)	Asian (AS)	Asia American (AA)
				All lineages	E-350-G				
[50]	115	115	L1	69%	Not Detected	-	15%	-	8%
[48, 51–54, 56]	621	106	E6	78%	29%	2%	2%	9%	4%
[49]	24	21	E6E7	71%	53%	-	-	-	-
[55]	205	21	LCR	42.8%	-	-	-	-	57.2%

(–) not evaluated; *AA* Asia-American HPV16 Variant; *AF* African HPV16 Variant; *AS* Asian HPV16 Variant; *E* European HPV16 variant; *E6* HPV Early protein 6; *E7* HPV Early protein 7; *LCR* long control region; *L1* late gene; *NA* North American HPV16 variant

As it was established from molecular analysis studies in cervical cancer, that the European variant T350G, was the variant frequently found in cervical intraepithelial neoplasms and cancers, and has been associated with progression to cervical cancer particularly in North European women. The detection of the T350G variant in a large proportion of HNSCC patients, therefore, indicates that this variant might also play an important role in HN carcinogenesis. Hassani et al.in 2015 supported this hypothesis, elucidating that the HPV-16 E-350G variant has a polymorphism in residue 83, a leucine for valine (L83V), probably responsible for the increased cancer risk [53].

## Conclusions

In HNSCC different lineages of HPV16 variants can be identified and differ geographically. Although most reviews described only the distribution of HPV16 variants in HNSCC, LeConte in a more detailed analysis of those studies found important differences. They found the distribution of two HPV16 variant groups differ significantly in oropharyngeal cancer and cervical cancer. The European + South America (E + AS) variant groups showed a higher prevalence in the oropharyngeal samples, representing 90.2%, than in cervical carcinomas (71.4%). Moreover, the Asia-American (AA1 + AA2) variant groups were present in 22.5% of cervical cancers in contrast to 4.4% in the oropharyngeal cancers [24]. The number of cases studied does not allow us to explore the importance of those differences nor to understand the potential role determining the clinical behaviour and potential use to select treatment and prognosis. Hopefully, the new sequencing era will enrich the study of HPV and related cancers. The advances in HPV whole-genome sequencing [46] provided technically achievable large-scale longitudinal studies on HPV whole-genomic sequences and promotes an exhaustive understanding of the viral genetic diversity within and between infected individuals and will make the link between variants and cancer risk, comprehensively [46, 61].

## Data Availability

Not applicable.

## Abbreviations

AA: Asia-American HPV16 variant
AF: African HPV16 variant
AS: Asian HPV16 variant
E: European HPV16 variant
E6: HPV Early protein 6
E7: HPV Early protein 7
HNSCC: Head and neck squamous cell carcinoma
HPV: Human papillomavirus
NA: North American HPV16 VARIANT
NGS: Next generation sequencing
